# Improving the Varroa (*Varroa destructor*) Control Strategy by Brood Treatment with Formic Acid—A Pilot Study on Spring Applications

**DOI:** 10.3390/insects13020149

**Published:** 2022-01-30

**Authors:** Eliza Căuia, Dumitru Căuia

**Affiliations:** Department of Honeybee Genetics and Breeding, Institute for Beekeeping Research and Development, Blvd. Ficusului No 42, Sector 1, 013975 Bucharest, Romania; dumitru.cauia@icdapicultura.ro

**Keywords:** brushing procedure, capped brood, formic acid, honey bee, spring application, varroa

## Abstract

**Simple Summary:**

The varroa mite control in a natural and sustainable way is critical for beekeeping, taking into account the importance of honey bees for pollination as well as for obtaining clean products. In recent time, new procedures for varroosis treatment in the reproductive phase were developed, which can be applied any time during the active season as they use volatile organic acids, widely accepted for organic beekeeping. Such a procedure consists of brushing the capped brood with formic acid, which is very effective in killing varroa mites but also minimally invasive for honey bee colonies. The importance of varroosis treatments before winter bee rearing is evident and widely accepted, as most of the actual treatments are limited to the late active season applications for different reasons, especially because they are focused on phoretic mites. Having in view the flexibility of the new procedure’s application in the whole period of the active season, we started a pilot study to preliminarily test the effectiveness of spring applications on varroa mite control. The results show significant differences in brood infestation between experimental and control groups, in the same apiary, which gives clear indications that spring applications could be beneficial for improving the varroa control strategies.

**Abstract:**

The importance of varroosis control in a natural and sustainable way is crucial for beekeeping, having in view the varroa mite impact on honey bee health. In the last years, we developed a highly effective procedure for treating varroa in capped brood using volatile organic acids. This procedure can be applied at any moment of the active season as it uses organic substances. Taking into account the necessity to drastically reduce the level of varroa infestation in colonies before winter bee rearing, we developed a relatively simple pilot study to preliminarily test the impact of spring treatments on varroa infestation level in brood, to be evaluated in summer when, naturally, the population of mites increases. To test the hypothesis, two experimentally treated groups and a control group were used. The treatment consisted of brushing all capped brood with formic acid of 65% concentration in one and two applications. The obtained results show very significant differences between the treated and control groups in terms of infested cell percentages evaluated in the July–August period. Consequently, the spring treatments could be an important tool in limiting the varroa mite multiplication, but further experiments are necessary to test and adapt them to different local conditions.

## 1. Introduction

It is well known that the *Varroa destructor* mite [1] causes significant damage in worldwide beekeeping, being considered a major factor [2,3,4,5,6] of why the honey bee (*Apis mellifera*) populations have declined in recent times [7]. The destructive effect is the result of feeding behaviour [8], but also of the transfer of specific viruses [6,9] in close connection with various factors (amount of brood, type of brood, duration of active season, inter-colonies transfer) that favour the increase of the infestation level [2,3,4,5]. The host–parasite relationship and its multiplication process in *Apis cerana* [10,11] show us that the defence mechanisms of this honey bee species against the varroa mite are based mainly on its population dynamics and on different, highly pronounced hygiene behaviours, the reproduction limitation to the drone brood, and even a very specific behaviour which leads to the sacrifice of one’s own offspring through the so-called entombing phenomenon of drone brood. All these are part of a very complex strategy, the result of an extremely long coevolution and an inter-specific adaptation phenomenon [10]. 

Unfortunately, *A. mellifera* does not have all these adaptive behaviours in such a complex way, being far less resistant [2,3,4]. The mite multiplication has much wider dynamics with a continuous growth of the population, which leads to the collapse of colonies in 1–3 years in the absence of treatments [2,4]. The reproduction of the varroa mite in *A. mellifera* honey bee, on which the success of its multiplication depends, takes place in the capped brood, both drone and worker brood [2,12,13]. The dynamics of the mite population during the active season is very variable and is based on a multiplication rate of 0.7–1.45 daughters per mature female in the worker brood and 1.6–2.6 daughters in the drone brood [2,3,12], in approx. 1.5–3 reproductive cycles on each mature female [2,14] and an infestation rate of 5 to 12 times higher in drone brood [2,3,12,15]. Different other natural factors (e.g., drifting, robbing, swarming, hygiene, the brood period, other local conditions, etc.) can accelerate or limit the whole process of multiplication [2,3,13,14,15,16,17,18].

As the brood caps are a barrier in the application of contact acaricides, and the current treatments are applied especially towards the end of the active season in order to kill phoretic mites, taking into account also the end of the main honey flows, the varroa mite population can reach significant thresholds that lead to winter bee damage and therefore to a general ineffectiveness of the control methods [2,19,20].

As a result, it is becoming increasingly important to approach new control strategies by non-polluting methods, which can be applied as early as possible in the season, thus targeting the reproductive phase as well [19,20,21,22,23,24,25]. In this sense, the application of volatile organic acids (e.g., formic and acetic acid) in short-term treatments on the whole colony [26,27] or brood [24,28,29,30,31] by various methods has been already proved useful.

Thus, in the treatment of the honey bee colony when formic acid is used, the level of efficacy in controlling the mite in parallel with the protection of the colony depends on the combination of concentration, exposure time and outside temperature [32,33,34,35]. Medium- and long-term treatments can have shortcomings caused by the risks on the viability of worker bees and queens or on the normal development of the honey bee colony as a whole [26,28,32,33,34]. 

In the brood treatment applied outside the colony, without bees, these risks are practically eliminated, as well as concentration-time-temperature variables [24,28,30,35]. Additionally, the brood offers constant parameters for volatilization [24]. As capped brood frames can be separated by colony during the application, the treatment by brood brushing with highly volatile acids-based liquid formulas is minimally invasive, being focused only on the infested brood, and is very flexible regarding time and method of application [24]. More than this, for high efficacy, the duration of the brood treatment can be limited to a much shorter duration (minutes) as compared with the treatment of the entire honey bee colony (at least 24 h) [24,26,27]. As a result of rapid volatilization properties of formic acid, for example, the impact on varroa mite mortality is very good (up to 100%) and immediate. 

Last but not least, formic acid, being an organic substance, does not pollute the hive products and does not contribute to increasing the resistance of the mite to the treatment substances, which support the applications throughout the whole active season for a continuous sustainable control [2,24].

Given the recent development of new treatment procedures [24,35] and the search for solutions to minimise the work load, we hypothesised that capped brood treatment performed as early as possible in the season (in spring, as early is possible) when there are smaller surfaces of brood, could significantly reduce the level of infestation in late summer, in the winter bee rearing period, which coincides with the period of maximum mite population development (August–September in temperate regions of Northern Hemisphere).

Consequently, the purpose of the work was to preliminary test, in a one-year pilot study, the effectiveness of the spring treatments, by brushing the capped brood with formic acid of 65% concentration, on varroa infestation level in the brood, assessed in the period when, naturally, the varroa population increases.

## 2. Materials and Methods

### 2.1. Experiment Design

#### 2.1.1. Biologic Material

Thirty honey bee colonies (*A.m. carpatica*) from the Honeybee Genetics and Breeding laboratory experimental apiary, belonging to Institute for Beekeeping Research and Development, were selected out of a larger pool of clinical healthy colonies, generally managed in Dadant hives on 10 frames. At the beginning of the experimental period (April 2021) the colonies were organised on 6–7 honey bee frames intervals and 3–4 brood frames, with young queens (July 2020) to assure the continuous egg laying in parallel with minimizing the swarming or robbing risks during the experimental period (April–August 2021). These experimental units were not treated against varroa mite one season before (end summer–autumn 2020) in order to dispose of biologic material (capped brood) with a higher level of infestation with varroa mite to highlight the effect of applied treatments.

#### 2.1.2. Tested Groups

The honey bee colonies were randomly grouped in 3 groups of 10 colonies each, as follows:

- Experimental group T1—for treatment of all capped brood in one application of formic acid of 65% concentration;
- Experimental group T2—for treatment of all capped brood in two applications of formic acid of 65% concentration at 10-day time interval, in order to treat the phoretic mites which would have entered in the newly capped brood;
- Control group T3—left untreated against varroa mite.

The experimental and control groups were placed randomly on the same location (44.491451, 26.086258), in 5 rows, with distances of ~1–1.5 m between colonies, thus being exposed to the same environmental conditions. All the colonies received the necessary management in order to assure the general conditions of nutrition and normal development. In the management of colonies, the brood and bee inter-colonies exchange was not accepted, but only food (frames with honey and beebread) exchange whenever it was necessary.

#### 2.1.3. Treatments

The first application of treatments in experimental groups was carried out on 6 April 2021 on 20 honey bee colonies assigned to T1 and T2 treatment groups, and a second application was applied on 16 April 2021 only on the 10 colonies assigned to T2 treatment groups. The working procedure consisted of brushing the capped brood surfaces with formic acid of 65% concentration, according to the described details in the literature [24].

#### 2.1.4. Measurements

In order to evaluate the varroa mite infestation level in capped brood and therefore the efficacy of treatments as compared with the control group, three measurements were performed in the July–August 2021 period at different time intervals. The measurements consisted of counting the infested cells in worker brood in the selected areas and expressing them as percentage of infested cells/frame (colony) which represents the response variable for all the statistical analyses. In our experiments we opted for this method of estimation of the mite population at the colony level which proved to be quite a precise method as compared with phoretic mite infestation [36,37,38]. 

In order to perform the measurements, a central capped brood frame per colony was taken out and examined in laboratory conditions at different time intervals (5–8 July; 20–22 July; 16–18 August). Every capped brood frame was selected to have capped brood on both sides. A total of 400 cells were examined on each frame, with 200 cells on each side. Each side of the brood frame was examined so that the measurements were performed on different smaller surfaces of brood (4 spots × 50 cells), randomly selected, in order to better represent the probability of finding the infested cells that sometimes are found as aggregated spots of infested cells. In the literature are described more methods for brood examination, the precision being correlated with the examined surfaces and repeated measures, but a minimum of 200 cells/colony was mentioned [37,38]. The combs’ examination was carried out under stereomicroscope (Olympus SZ61) at minimum 6.7X magnifications in order to clearly observe the infested cells. The capped cells were opened with tweezers, cell by cell, in more rows. The infested cells were more easily identified by the presence of white mite dejection spots on the cell walls, but each pupa inside the examined cells was taken out to confirm the presence of mites. 

#### 2.1.5. The Statistical Analysis

The obtained data were statistically analysed and interpreted by using NCSS 2021 v21.0.2 software with data normality, variance and means homogeneity assumption tests, applied on different sets of data, being completed by post hoc tests when significant differences were registered, according to software recommendations and literature [39,40,41].

## 3. Results 

The obtained results regarding the percentage of infested brood in different colonies, treatments and periods of evaluation are presented in Table S1 and Figure S1a,b in the Supplementary Material. Out of these data the results of the level of varroa mite infestation in brood on the whole period was:

- Between 0.25% and 6.5% infested cells with an average of 2.46% in the T1 experimental group—one application;
- Between 0.25% and 4.5% infested cells with an average of 1.92% in the T2 experimental group—two applications at a 10-day interval;
- Between 2.75% and 15.5% infested cells with an average of 7.74% in the T3 control group left untreated.

**Figure 1:**
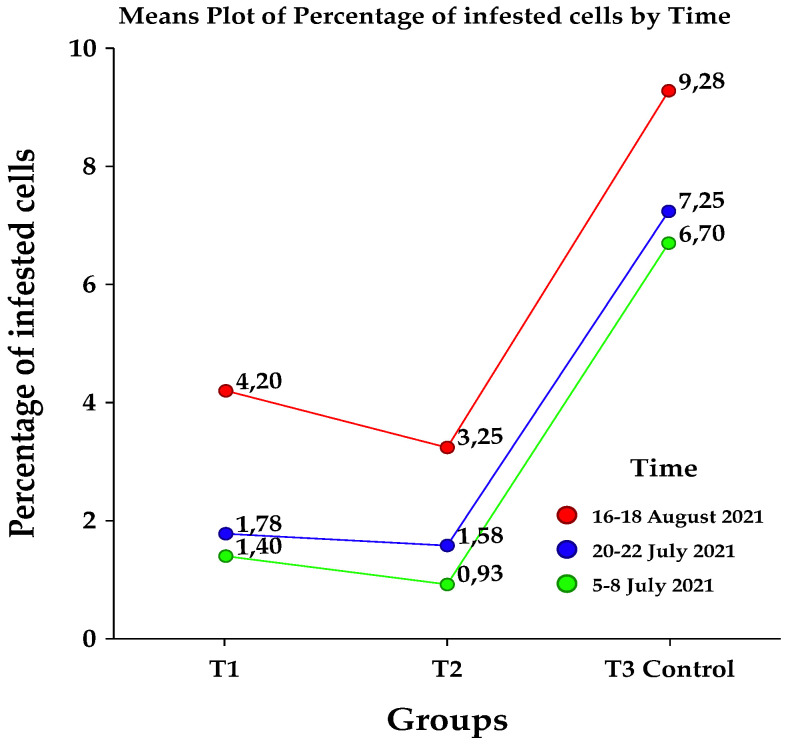
The mean percentage of varroa mite infested cells evaluated in brood at different time intervals, as well as in different experimental groups.

In order to have a simplified image on the obtained data sets, a statistical summary is presented in Table 1, the mean values being plotted in the Figure 1. 

**Figure 2:**
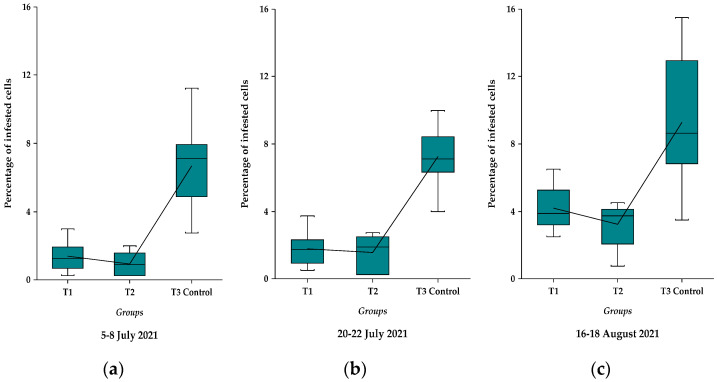
The box plotted data on the percentage of varroa mite infested cells evaluated in different experimental groups (T1, T2) as compared with T3 control in different time intervals: (**a**) 5–8 July 2021; (**b**) 20–22 July 2021; (**c**) 16–18 August 2021. The lines between treatments unite the mean values of each treatment and the central horizontal lines represent the median values.

In order to highlight the differences between treatments so to have a better overview on the impact of treatments on the level of infestation evaluated at different time intervals in the summer (July and August), the results were statistically analysed using different tests for data normality, outliers, variance, and means homogeneity [39,40,41]. The results on different periods of evaluations are also plotted and presented in Figure 2a–c. 

Using the normality tests (Shapiro–Wilk, Anderson–Darling) for each set of data based on groups and time interval as break variables, the results show that the percentage of varroa mite infested cell data are normally distributed (α = 5%), so the parametric statistics tests will be used further in the statistical analysis. 

The Grubbs test shows that there are no outliers values when the analyses are performed on each set of data, as it can be noticed in the Figure 2a–c. When the data are analysed together, then there are no outliers in the T1 and T2 experimental groups, but two higher values (15.5%, 15%) are displayed in the T3 Control, 16–18 August 2021 set of data as possible outliers. The highest value (15.5%) is also highlighted by a dot in Figure S1a in the Supplementary Material. These possible outliers appear as a normal consequence of high levels of infestation due to lack of treatment, especially at the end of the season, when the mean percentage reached 9.28% (Table 1), and therefore it was decided to keep them in the statistical analysis.

Levene’s test used at confidence level of α = 0.05 shows that variances are not homogeneous in the two data sets collected in 5–7 July and 16–18 August, but are homogenous in the data set collected in 20–22 July (Table 2). Based on these different results, the variance equality assumption was also tested at a more precise confidence level (α = 0.001). The new, refined results show that the variances are homogenous in all data sets. Due to these possible statistical differences, the following analyses were also performed at both levels of confidence. 

In order to test the means homogeneity assumption, the next tests in statistical analysis were used: the Welch’s ANOVA test assuming the unequal variances (Table 3) and the one-way ANOVA statistical test assuming the equal variances (Table 4). These tests were followed by the Tukey–Kramer post hoc test to establish the significance of the differences between treatments calculated on mean values (Table 5).

The Welch’s ANOVA test applied on data with unequal variances (Levene’s test at α = 0.05) shows highly significant differences (F = 24.99; α = 0.001; *p* = 0.000) between the mean values of tested groups for data sets collected in the 5–7 July period, as well as significant differences but not highly significant (F = 9.81; α = 0.001; *p* = 0.002) between the tested groups for data sets collected in the 16–18 August period.

The one-way ANOVA test allowing equal variances (Levene’s test at α = 0.001) shows highly significant differences (F = 43.02; F = 60.79; F = 15.79; α = 0.001; *p* = 0.000) between the means of different groups in all periods of collecting data.

Following application of the Tukey–Kramer multiple-comparison test, the results (Table 5) show highly significant differences between the percentage of infested cells of each treated group (T1 and T2) as compared with T3, the control group, as calculated values (6.7; 7.25; 9.2) in the control group are greater than the critical value (5.78) at α = 0.001. 

The same statistical results also show that between the two experimental groups (T1 as compared with T2) there are no significant differences in the data collected in the July period, but the differences appear as significant in August (calc. value = 4.2 > crit. value = 3.51; α = 0.05), but not very significant (calc. value = 4.2 < crit. value = 5.78; α = 0.001).

As mentioned before, to have a better overview on data sets for the different groups and evaluation periods, the results regarding the percentage of the infested cells are plotted in the Figure 2a–c. 

Out of these figures one can easily notice the differences between the experimental groups (T1 and T2) as compared with control group (T3) in every evaluation period, being demonstrated also by statistical analyses. The differences between July and August months, when the infestation level increased substantially, are also notable.

In the experimental groups (T1 and T2), the lowest infestation levels were obtained in the T2 variant, in which two treatments were repeated at a 10-day interval. The non-significant differences between the two experimental groups in July, but significant in August, show that there are notable differences between the two variants of treatments in spring applications. 

## 4. Discussion

The very significant differences between the treated colonies and control ones (untreated) in terms of varroa mite infested cells percentage in capped brood indicate that spring treatments in one or two applications affect the mite population development throughout the year, confirming the study hypothesis. 

These results offer an important image of the impact of initial varroa load on summer mite population in the treated and untreated worker brood. Consequently, the study demonstrates that management of varroosis in spring is very important for the year-round strategy control of the mite population. 

The significant differences between the two experimental treatments (T1 and T2) in varroa infestation found at the end of the season (August) show the importance of minimizing the level of infestation at the beginning of the season.

In a previous study [21], the mite infestation level in summer evaluated on adult bees was significantly reduced by early spring bio-technical management, especially when early brood interruption technique combined with an oxalic acid treatment was used. 

Unfortunately, the actual strategies on varroa control are focused on phoretic mites in summer–autumn treatments. This is because the level of infestation in spring is low and is based on residual mites from the previous season, being found mainly in brood in the reproductive phase. The mite population in spring is not critical for spring and summer colony development but is critical for the end of the season when winter bees are reared and damages are usually observed.

It is well known that the natural transfer of *V. destructor* between colonies can take place through robbing and drifting of drones and worker bees by homing errors [18]. Bees’ drifting between colonies from the same apiary or between different apiaries is a common phenomenon and data show a large variability (0–96%) depending on the individual (worker or drone), colony physiology, distance between hives, position, hive painting, level of infestation, wind direction, landmarks, number of colonies, etc. [18,42,43]. 

In this context, the infestation level identified in the brood of colonies from experimental variants could be the result of natural multiplication of residual mites after the treatment application and/or the inter-colonial transfer especially by drifting phenomena, as robbed colonies were not noticed in the control group during the experimental period. However, the robbing could be a possible route of mite inter-colonial transfer if robber worker bees from experimental colonies went outside the experimental apiary. It is also possible that other unknown factors could be involved in the mite inter-colony exchange.

Out of the obtained results one can notice also a slightly lower brood infestation percentage in the experimental group who received two treatments at a 10-day interval as compared with one application, but the differences between these two experimental groups were statistically significant only in August evaluation period. These significant differences between the two experimental groups in August are probably the result of different single or combined multiplication scenarios:

- The multiplication of varroa in worker brood;
- The multiplication of varroa in drone brood in the previous months and their transfer into the worker brood as a result of drone brood growth cessation, which coincide with the end of July–beginning of August in the experimental location;
- The reinfestation by drifting;
- The reinfestation via the robber honey bees from the experimental colonies.

In our study, the drifting phenomena could explain some of the level of infestation found in experimental groups, especially in the T2 group, as a result of the random and close arrangement of experimental groups together with control groups on the same apiary location [18]. 

Therefore, a clearer difference between the experimental groups and control groups would be better highlighted by avoiding honey bee drifting (the exchange of mites) by placing treated colonies at larger distances (≥1.5 km) from infested groups, as the literature indicate [43]. In our case, we can only anticipate that the difference between the infestation levels in the experimental groups as compared with the control group would have been greater in absence of drifting. Thus, from a practical point of view, it appears to be very important that an effective and sustainable control of *V. destructor* in an apiary depends on treatment effectiveness at each level: colony, apiary, and colonies/apiaries in the honey bee flight area.

The estimation of the mite infestation at brood level in the chosen period of time offers an interesting image when data obtained in July are compared with August ones, which show an increase in the infestation level in experimental colonies treated once. These differences could be the result of the drone brood rearing ceasing and, consequently, of the increasing of infestation in worker brood, which usually happens at the end of July–beginning of August (in the southern area of Romania), combined with the drifting phenomenon as well. 

As the present research represents a pilot study which indicates that there are significant differences between treated and untreated colonies, the experimental conditions and infestation level measurements could be further improved and applied at a larger scale in different local conditions to improve the results and draw the best conclusions and recommendations in local varroa control. 

Increasing the optimal time interval for applying treatments, including early season applications by brood treatments, particularly using brushing procedures with highly volatile organic acids, a better effectiveness in *V. destructor* mite control is obtained. Thus, the development of critical thresholds of varroa mite population before and during the winter bee rearing period is minimised, an extremely important element in maintaining the honey bee longevity during the winter, so of a better colony health.

## 5. Conclusions

The main conclusion of the present study is that the varroosis control by spring treatments applied on brood is very effective in reducing the mite population in summer. This treatment could be an important tool to be included in further studies and varroosis control strategies adapted to local conditions, with minimal costs and risks, and maximum advantages on honey bee colony health.

## Figures and Tables

**Table 1:** The basic descriptive statistics of data regarding the percentage of varroa mite infested cells evaluated at different time intervals, in the experimental and control groups.

Data Sets	Descriptive Statistics of Data Sets (Percentage of Infested Cells)						
	Mean	St. Dev	St. Err.	Min.	Max.	Counts (Colonies)	Counts (Cells)
Groups|Time Interval of Data Collection							
T1|5–8 July	1.40	0.87	0.27	0.25	3.00	10	400
T1|20–22 July	1.78	0.95	0.30	0.50	3.75		400
T1|16–18 August	4.20	1.31	0.41	2.50	6.50		400
T2|5–8 July	0.93	0.65	0.20	0.25	2.00	10	400
T2|20–22 July	1.58	1.01	0.32	0.25	2.75		400
T2|16–18 August	3.25	1.30	0.41	0.75	4.50		400
T3 Control|5–8 July	6.70	2.45	0.77	2.75	11.25	10	400
T3 Control|20–22 July	7.25	1.79	0.57	4.00	10.00		400
T3 Control|16–18 August	9.28	4.07	1.29	3.50	15.50		400

**Table 2:** The analysis of data variance applied on different data sets of treatments (groups) and evaluation time intervals at significance level 0.05 and 0.001.

Levene’s Test		Test Value	Probability Level	Reject Equal Variances? α = 0.05	Reject Equal Variances? α = 0.001
1	5–7 July 2021	6.389	0.005	Yes	No
2	20–22 July 2021	2.052	0.148	No	No
3	16–18 August 2021	6.491	0.005	Yes	No

**Table 3:** The Welch’s ANOVA test of means, allowing for unequal variances of the different treatments grouped on the evaluation time interval.

Welch’s ANOVA Test of Means Allowing for Unequal Variances	Numerator DF	Denominator DF	F-Ratio	Probability Level	Reject Equal Means? (α = 0.05)	Reject Equal Means? (α = 0.001)
Between treatments evaluated on:						
5–7 July 2021	2	16.03	24.99	0.000	Yes	Yes
16–18 August 2021	2	16.50	9.81	0.002	Yes	No

**Table 4:** The one-way ANOVA test of means allowing for equal variances of the different treatments grouped on the evaluation time interval.

One-Way ANOVA Test of Means Allowing Equal Variances	DF	Sum of Squares	Mean Square	F-Ratio	Probability Level	Reject Equal Means? (α = 0.05 & 0.001)
Between treatments evaluated on:						
5–7 July 2021	2/27	205.5	102.7	43.02	0.000	Yes
20–22 July 2021	2/27	207.4	103.7	60.79	0.000	Yes
16–18 August 2021	2/27	209.8	104.9	15.79	0.000	Yes

**Table 5:** The Tukey–Kramer multiple-comparison test on the different treatments and different time intervals.

Between Treatments		Evaluated on		
		5–7 July	20–22 July	16–18 August
Groups	Counts	Means		
T-1	10	1.4	1.78	4.2
T-2	10	0.93	1.58	3.25
T3 Control	10	6.7	7.25	9.28
Critical value = 3.51 (α = 0.05), critical value = 5.78 (α = 0.001)				

## Supplementary Materials

The following supporting information can be downloaded at: https://www.mdpi.com/article/10.3390/insects13020149/s1, Table S1: The percentage of infested cells with the varroa mite in the experimental and control groups and different periods, following the brood treatment with formic acid 65% applied by brushing procedure. Figure S1a: A general overview of the box plotted data on the percentage of varroa mite infested cells evaluated in different experimental groups (T1, T2, T3 control) in the whole evaluation period, July–August 2021. Figure S1b: The mean percentage of varroa mite infested cells evaluated in different experimental groups (T1, T2, T3 control) in the whole evaluation period, July–August 2021.
