# Supplementary material for: Improving the Varroa (Varroa destructor) Control Strategy by Brood Treatment with Formic Acid—A Pilot Study on Spring Applications
Source: Insects. 2022 Jan 30;13(2):149. doi: 10.3390/insects13020149 (PMC8875234; doi:10.3390/insects13020149)
Supplement: Supplementary file 1 [file insects-13-00149-s001.zip › insects-1553120-supplementary.pdf]

insects

MDPI

Table S1. The percentage of infested cells with the varroa mite in the experimental and control groups and different periods, following the brood treatment with formic acid 65% applied by brushing procedure.

**Groups**	**The percentage of infested cells**								
	T1			***T2***			***T3 Control***		
**Evaluated colonies & periods**	*5-8 July 2021*	*20-22 July 2021*	*16-18 August 2021*	*5-8 July 2021*	*20-22 July 2021*	*16-18 August 2021*	*5-8 July 2021*	*20-22 July 2021*	*16-18 August 2021*
**1**	1.75	2.25	3.5	0.25	0	1.5	9.25	4	4
**2**	1.25	0.5	2.5	1.75	2.5	3.75	7.25	8	15.5
**3**	0.25	0.75	4.25	1	0.25	2.25	2.75	5.75	7.75
**4**	2.5	1.75	4.5	0.25	2.25	4.5	4.5	7.75	9.5
**5**	1.25	3.75	6	1.5	1.75	4.5	11.25	9.75	15
**6**	1.75	1.5	3.5	0.25	2	4	7.25	10	7.75
**7**	0.5	1.75	3	1	1.25	3.75	7	6.75	3.5
**8**	0.75	1	3.25	0.5	0.25	0.75	5	6.5	9.75
**9**	1	2	5	2	2.75	4	5.25	7.5	12.25
**10**	3	2.5	6.5	0.75	2.5	3.5	7.5	6.5	7.75

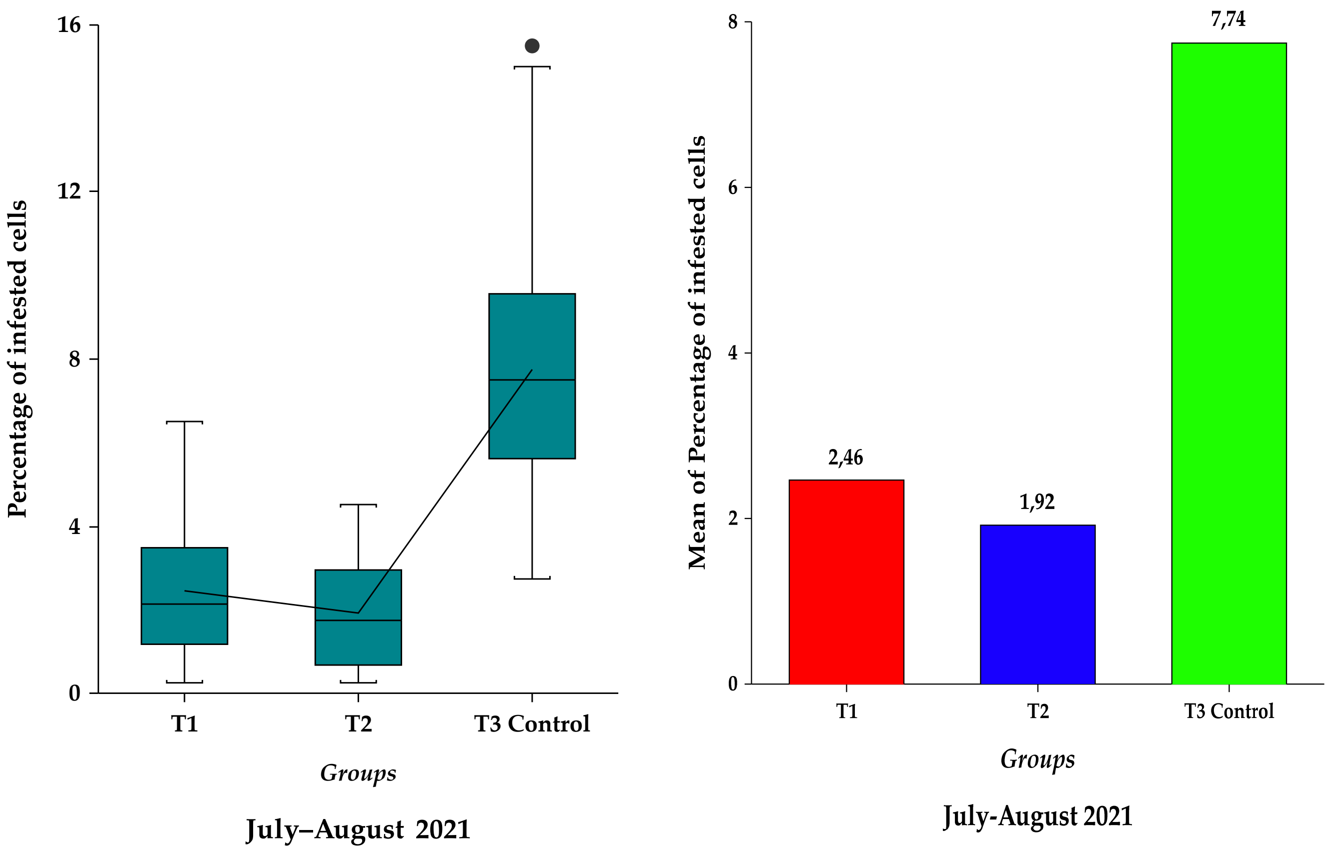

(a) **(b)**

Figure S1 (a): A general overview of the box plotted data on the percentage of varroa mite infested cells evaluated in different experimental groups (T1, T2, T3 Control) in the whole evaluation period: July-August 2021. **(b)** The mean percentage of varroa mite infested cells evaluated in different experimental groups (T1, T2, T3 Control) in the whole evaluation period: July-August 2021.
